# Metabolomics Analyses Provide Insights Into Nutritional Value and Abiotic Stress Tolerance in Halophyte *Halogeton glomeratus*

**DOI:** 10.3389/fpls.2021.703255

**Published:** 2021-07-05

**Authors:** Juncheng Wang, Ke Yang, Lirong Yao, Zengke Ma, Chengdao Li, Erjing Si, Baochun Li, Yaxiong Meng, Xiaole Ma, Xunwu Shang, Huajun Wang

**Affiliations:** ^1^Gansu Provincial Key Lab of Aridland Crop Science/Gansu Key Lab of Crop Improvement and Germplasm Enhancement, Lanzhou, China; ^2^Department of Crop Genetics and Breeding, College of Agronomy, Gansu Agricultural University, Lanzhou, China; ^3^Western Barley Genetics Alliance, College of Science, Health, Engineering and Education, Murdoch University, Murdoch, WA, Australia; ^4^Department of Botany, College of Life Sciences and Technology, Gansu Agricultural University, Lanzhou, China

**Keywords:** *H. glomeratus*, metabolomic, secondary metabolites, pharmacological effects, abiotic stress

## Abstract

*Halogeton glomeratus* is a succulent annual herbaceous halophyte belonging to the Chenopodiaceae family, has attracted wide attention as a promising candidate for phytoremediation and as an oilseed crop and noodle-improver. More importantly, *H. glomeratus* has important medicinal value in traditional Chinese medicine. However, there are few comprehensive studies on the nutrients, particularly secondary metabolites. Here, we adopted untargeted metabolomics to compare the differences in metabolites of different tissues (root, stem, leaf, and seed) and identify the compounds related to pharmacological effects and response to abiotic stress in *H. glomeratus.* A total of 2,152 metabolites were identified, and the metabolic profiles of root, stem, leaf, and seed samples were clearly separated. More than 50% of the metabolites showed significant differences among root, stem, leaf, and seed. The Kyoto Encyclopedia of Genes and Genomes (KEGG) pathway enrichment analysis of differential metabolites suggested an extensive alteration in the metabolome among the different organs. Furthermore, the identified metabolites related to pharmacological effects and response to abiotic stress included flavones, flavonols, flavandiols, glucosinolates, isoquinolines, pyridines, indoles, amino acids, lipids, carbohydrates, and ATP-binding cassette transporters. These metabolites have application in treating human cardiovascular diseases, cancers, diabetes, and heart disease, induce sleeping and have nutritive value. In plants, they are related to osmotic adjustment, alleviating cell damage, adjusting membrane lipid action and avoiding toxins. To the best of our knowledge, this is the first metabolomics-based report to overview the metabolite compounds in *H. glomeratus* and provide a reference for future development and utilization of *H. glomeratus*.

## Introduction

Metabolomics, a rapidly developing science and technology system of comprehensive and simultaneous analysis of biological sample metabolite profiles, has become routinely applicable across plants, humans, animals, and bacteria (Schauer and Fernie, 2006; Okada et al., 2010). In the plant research field, metabolite profiling was first applied as a diagnostic tool to determine barley seedling response to herbicides (Sauter et al., 1991). It has gradually provided a deep understanding of the classification of plant genotypes (Fiehn et al., 2000; Matsuda et al., 2012), crop breeding (Fernie and Schauer, 2009; Alseekh et al., 2018), phytochemical genomics (Okazaki and Saito, 2016), and deciphering gene function (Quanbeck et al., 2012; Li and Song, 2019; Szymański et al., 2020) and responses related to environmental (Shulaev et al., 2008; Feng et al., 2019) and/or genetic perturbations (Dixon et al., 2006; Fraser et al., 2020).

Plant metabolites involve a wide variety of chemical compounds with diverse biological functions, and the number is estimated to exceed 200,000 (van der Kooy et al., 2009). Specialized secondary metabolites from plants serve as rich resources for drug, food and nutraceutical development. Plant bioactive compounds such as polyphenols, carotenoids, glucosinolates, alkaloids, and terpenes are known to have important health benefits (Cicero and Colletti, 2017). Polyphenols can effectively prevent cardiovascular diseases, cancers, neurodegenerative diseases, diabetes, osteoporosis, and oral disease due to high antioxidant or anti-inflammatory activity (Scalbert et al., 2005; Ferrazzano et al., 2011). The classification of polyphenols in plants mainly includes chalcones, flavones, flavonols, flavandiols, anthocyanins, proanthocyanidins, and aurones (Winkel-Shirley, 2001). Acetylsalicylic acid, known as aspirin, is the major salicylate in the willow tree (*Salix* sp.) (Mahdi et al., 2006). Tropane alkaloids, commonly used as anticolic and spasmolytic drugs, are secondary metabolites enriched in Solanaceae plants (Śramska et al., 2017). Many coloring, flavoring, texturizing, preservative and anti-browning agents are secondary metabolites produced in plants (Kallscheuer et al., 2019). Several plant flavonoids, alkaloids, terpenoids, lignins, tannins, and tocopherols have gained scientific interest due to their health effects in helping to prevent diseases, improve immune response and provide antioxidant activity (Nirmala et al., 2020).

Halophytes, plants that exhibit efficient salt-tolerance mechanisms to cope with salt stress, constitute about 1% of the world’s flora (Flowers and Colmer, 2008). Potential applications of halophytes have been recognized for phytoremediation, desalination, vegetables, forage, oilseed crops, secondary metabolite production, medicine, and saline agriculture (Glenn et al., 2013; Nikalje et al., 2018). In recent years, metabolite profiling has been performed on a diverse array of halophytes including analysis of tolerance to osmotic stress (Lugan et al., 2010), response to salt stress (Wang et al., 2021), toxicological effects of environmentally relevant concentrations of heavy metals (Liu et al., 2011; Wu et al., 2012), and plant nutritional potential (Mishra et al., 2015; Li and Song, 2019). The untargeted metabolomics approach has a great advantage in the identification of known or unknown chemically diverse metabolites, which is more suitable for the assessment of potential biomarkers or metabolic mechanisms of plants (Hasanpour et al., 2020).

*Halogeton glomeratus*, a succulent annual herbaceous halophyte belonging to the Chenopodiaceae family, is widely distributed in Central Asia and arid regions of northwestern China due to its robust tolerance to drought and salt stress (Khan et al., 2001; Wang et al., 2015). This species is a promising candidate for phytoremediation of salt-affected and heavy metal-contaminated soils and as an oilseed crop (Abideen et al., 2015; Li et al., 2019). Its succulent leaves can hyperaccumulate salts and heavy metals, such as Na, K, Ni, Cu, Zn, and As (Li et al., 2019). The oil content of seeds is 19.2% and includes 91.8% unsaturated fatty acids, especially rich in linoleic and oleic acids (Li et al., 2019), and can effectively prevent the development of cardiovascular diseases, reduce body fat, modulate immune and inflammatory responses, and improve bone mass (Kim et al., 2016; Hasanpour et al., 2020). Furthermore, the salt extraction of *H. glomeratus* can improve the stretching quality of noodles, which is an important raw material source for additives in traditional Lanzhou beef noodles (Yan, 2015). More importantly, *H. glomeratus* has been prescribed in traditional medicine for the pharmacological functions of diaphoresis, relieving cough and asthma and dispelling dampness in oriental countries since ancient times. Therefore, *H. glomeratus* is undoubtedly an important plant resource with many uses. However, relevant research has only involved seed characteristics (Williams, 1960; Khan et al., 2001, 2009), species invasion (Duda et al., 2003), poisoning in livestock (James and Butcher, 1972; Lincoln and Black, 1980), heavy metal (Yan et al., 2011) and salt stress responses (Wang et al., 2015, 2018), and phytoremediation (Li et al., 2019). There have been few comprehensive analyses of metabolites in this species, especially secondary metabolites and its difference in different tissues, are not detailed. The poisonous nature of *H. glomeratus* to sheep grazing due to a large amounts of oxalic acid in plants (James and Butcher, 1972). Kudekova et al. (2017) provided a description on some biologically active compounds of this species using roots and aerial parts of *H. glomeratus*, including vitamin, amino- and fatty-acid compositions. In the present study, we adopted untargeted metabolomics to identify the compounds and compared the metabolite compositions of different *H. glomeratus* tissues (root, stem, leaf, and seed) to provide basic data for the future development and utilization of this species as a source of medicinal products and genes of resistance to abiotic stress.

## Materials and Methods

### Plant Material and Sample Preparation

As a traditional Chinese medicine, the whole plant of *H. glomeratus* is usually collected in August to October. Here, in mid-August and October 2020, a total of 24 whole robust plants of flowering period and mature seeds of *H. glomeratus* were randomly collected from Zhongchuan town, Lanzhou New District, in Gansu Province of Northwestern China (36°65’95.3”N,103°64’79.4”E), respectively. The town has a mid-temperate semiarid continental climate with annual mean rainfall of 300–350 mm, annual mean evaporation of 1,880 mm and annual mean air temperature of 6.9°C, as well as frequent drought. The soil is mostly loess and total salt concentration is 1.0% with pH of 8.1 at 0–40 cm depth in the soil profile. Furthermore, the organic matter is 19.2 g kg^–1^ and total nitrogen (N), phosphorus (P), potassium (K) are 1.25, 0.94, and 14.7 g kg^–1^, respectively. The available N, P, and K are 42.4, 14.8, and 205 mg kg^–1^, respectively.

**FIGURE 1:**
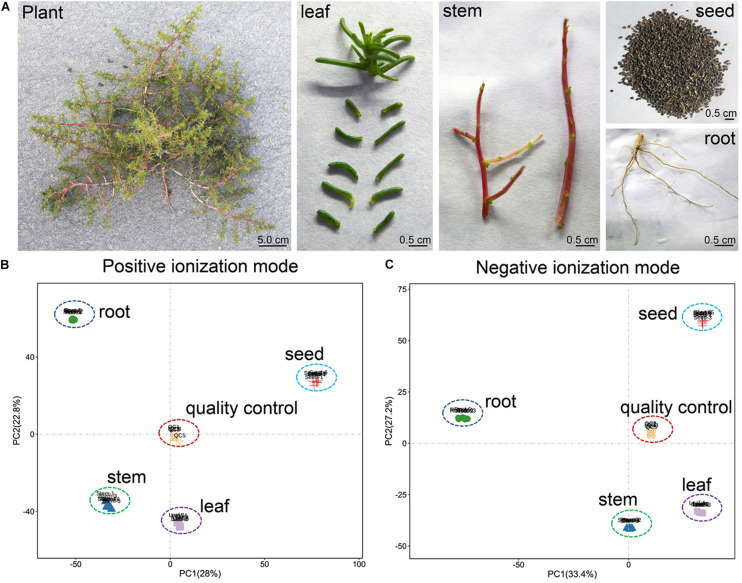
**(A)** Phenotype of *H. glomeratus* plant, leaf, stem, root and seed, and overall score plots of the PCA model with quality control in **(B)** positive and **(C)** negative ionization modes.

To better understand the metabolites composition and compare its differences in different tissues of *H. glomeratus*, the plants were taken to the laboratory and cleaned with distilled water and then divided into roots, stem, leaves, and seeds. The roots, stem, leaves, and seeds of four individuals were blended to form a composite sample with six replicates (Figure 1A). All samples were frozen in liquid nitrogen immediately and stored at −80°C until needed.

### Sample Extraction

The metabolites were extracted from the samples according to Ying et al. (2020). Firstly, freeze-dried samples were ground to powder using a grinder at 30 Hz for 1.5 min. Then 1 mL of pre-chilled methanol (−20°C) was transferred into 100 mg of lyophilized powder and vortexed for 1 min. Then, the sample was centrifuged at 20,000 *g* for 10 min at 4°C and the supernatant was absorbed and concentrated to dryness by vacuum concentrator (5305, Eppendorf, Germany). Samples were dissolved in 2-chlorobenzalanine (4 ppm) 80% methanol solution (−20°C) and the supernatant filtered through 0.22-μm micropores before liquid chromatography–tandem mass spectrometry (MS/MS) analysis. The quality control (QC) sample was prepared by taking 20 μL from each sample extract and used to monitor the reproducibility of the samples from the same organ.

### Detection of Metabolites

All steps of the ultra-performance liquid chromatography (UPLC) (Thermo UltiMate 3000) and MS/MS (Thermo Q Exactive) analyses were performed as described previously (Sanchez-Arcos et al., 2019). Briefly, 5 mL of each sample extract was injected into an ACQUITY UPLC^®^ HSS T3 (150 mm × 2.1 mm, 1.8 μm, Waters) column maintained at 40°C. The temperature of the autosampler was 8°C. Gradient elution of analytes was carried out with 0.1% formic acid in water (C) and 0.1% formic acid in acetonitrile (D) or 5 mM ammonium formate in water (A) and acetonitrile (B) at a flow rate of 0.25 mL/min. Injection of 2 μL of each sample was done after equilibration. An increasing linear gradient of solvent B (v/v) was used as follows: 0–1 min, 2% B/D; 1–9 min, 2–50% B/D; 9–12 min, 50–98% B/D; 12–13.5 min, 98% B/D; 13.5–14 min, 98%-2% B/D; and 14–20 min, 2% D-positive model (14–17 min, 2% B-negative model).

Mass spectrometry experiments were executed on a Thermo Q Exactive mass spectrometer with spray voltage of 3.8 and −2.5 kV in positive and negative modes, respectively. Sheath gas and auxiliary gas were set at 30 and 10 arbitrary units, respectively. The capillary temperature was 325°C. The analyzer scanned a mass range of m/z 81–1,000 for full scan at a mass resolution of 70,000. Data dependent acquisition MS/MS experiments were performed with HCD scan. The normalized collision energy was 30 eV. Dynamic exclusion was implemented to remove some unnecessary information in MS/MS spectra.

### Identification of Metabolites

Raw data files were converted to mzXMLformat using Proteowizard (v3.0.8789) and then the R statistical package XCMS (v3.1.3) was employed for data formatting, peak identification, filtration and alignment as described by Tautenhahn et al. (2012). Metabolites were analyzed by spectrum match to databases, including the Human Metabolome Database (HMDB),^1^ METLIN,^2^ Massbank,^3^ LipidMaps,^4^ and mzClound.^5^

### Statistical Analysis

To determine how the metabolic profiles differed among root, stem, leaf and seed, the identified metabolites were subjected to principal component analysis (PCA), partial least squares discriminant analysis (PLS-DA), and orthogonal projection to latent structures-discriminant analysis (OPLS-DA) using the corresponding R package models.^6^ The variable importance for projection (VIP) score from the (O)PLS model was applied to rank the metabolites that best distinguished between organs. Metabolites between two organs with VIP ≥ 1 and *t*-test *P* < 0.05 were used as a univariate analysis to identify for differential metabolites and then mapped to Kyoto Encyclopedia of Genes and Genomes (KEGG) metabolic pathways for pathway enrichment analysis (FDR ≤ 0.05). Hierarchical cluster analysis (HCA) was performed on the accumulation patterns of metabolites between different tissues using Pearson correlation distance matrix.

## Results

### Difference in Constitutive Metabolic Profiles of Root, Stem, Leaf, and Seed Samples

We conducted PCA to monitor the accuracy and reproducibility of the analysis process of root, stem, leaf, seed, and QC samples. Biological replicates of each sample were always grouped together with small confidence intervals. The first principal component (PC1) and the second principal component (PC2) explained 28 and 22.8% of the total variability for positive ionization mode (PI) datasets, respectively (Figure 1B). Similarly, PC1 and PC2 explained 33.4 and 27.2% of the total variability for negative ionization mode (NI) datasets, respectively. For both ionization modes, the metabolic profiles of root, stem, leaf, and seed samples were separated distinctly along both PC1 and PC2 (Figure 1C).

### Qualitative and Quantitative Metabolites

**FIGURE 2:**
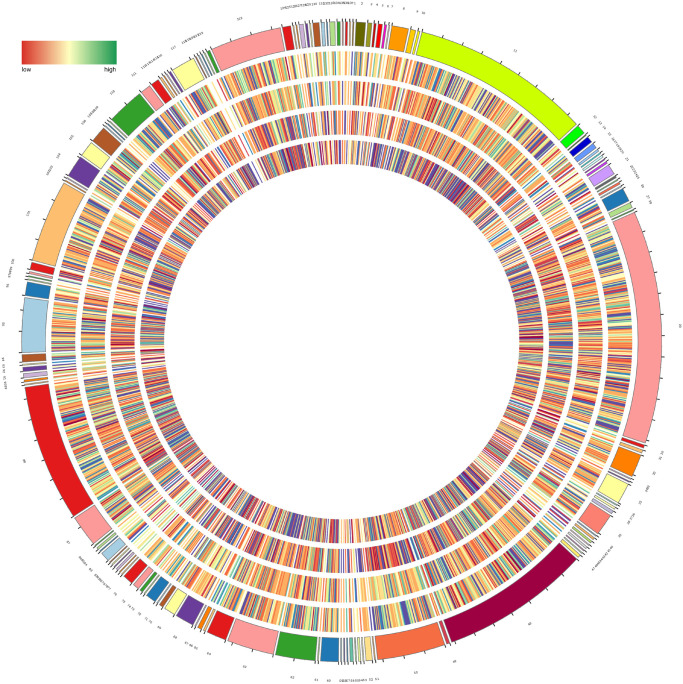
Circular diagram of all metabolites in *H. glomeratus.* Metabolites are classified into 137 categories and details are shown in Supplementary Table 2. From the outer to the inner circle are the metabolites of leaf, stem, root, and seed in each category, respectively. Color represents abundance of metabolites and the outermost circle represents quantity of each category.

A total of 2,152 known metabolites were identified based on widely targeted metabolites method, including 1,364 and 788 for PI and NI, respectively (Supplementary Table 1). It was necessary to note that 10,393 unknown metabolites were identified. All known metabolites were classified into 137 categories, with the dominant categories being carboxylic acids and derivatives, benzene and substituted derivatives, fatty acyls, organooxygen compounds, prenol lipids, steroids and steroid derivatives, glycerophospholipids, phenols, indoles and derivatives, and pyridines and derivatives (Figure 2 and Supplementary Table 2).

**FIGURE 3:**
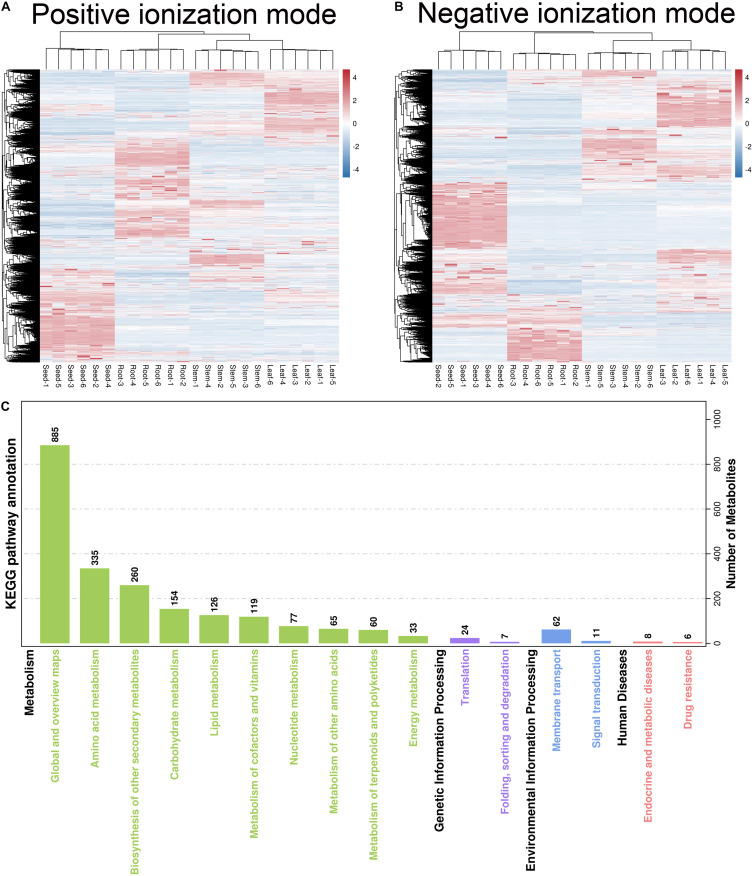
**(A,B)** Hierarchical cluster analysis and **(C)** KEGG pathway enrichment analysis of all identified metabolites. The heat map shows differential expression of metabolites between root, stem, leaf, and seed in **(A)** positive and **(B)** negative ionization modes. Red and blue color represent up-regulated and down-regulated metabolites according to log_2_ fold-change expression value, respectively.

Hierarchical cluster analysis was used to evaluate differences in accumulation patterns of metabolites between different *H. glomeratus* tissue samples. The sample classes were separated into four non-overlapping clusters, based on organs (Figures 3A,B), indicating that metabolite accumulation was organ-specific. The production of metabolites was mainly enriched in global and overview maps (885 metabolites), amino acid metabolism (335), biosynthesis of other secondary metabolites (260), carbohydrate metabolism (154), lipid metabolism (126), and metabolism of cofactors and vitamins (119) (Figure 3C).

### Identification of Differential Metabolites Among Organs

**FIGURE 4:**
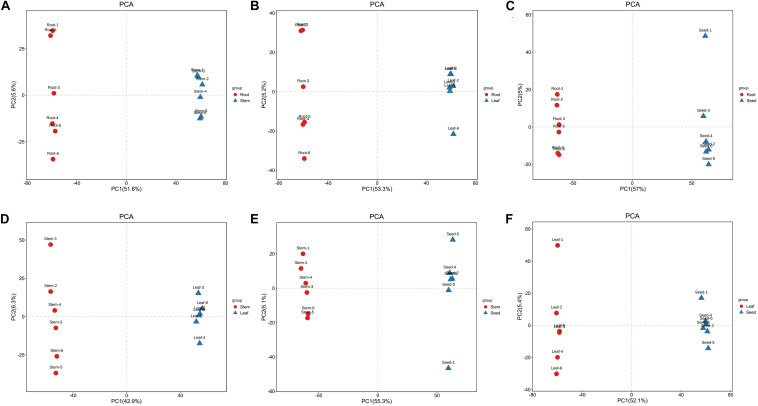
PCA score map of **(A)** root vs. stem, **(B)** root vs. leaf, **(C)** root vs. seed, **(D)** stem vs. leaf, **(E)** stem vs. seed, and **(F)** leaf vs. seed.

**FIGURE 5:**
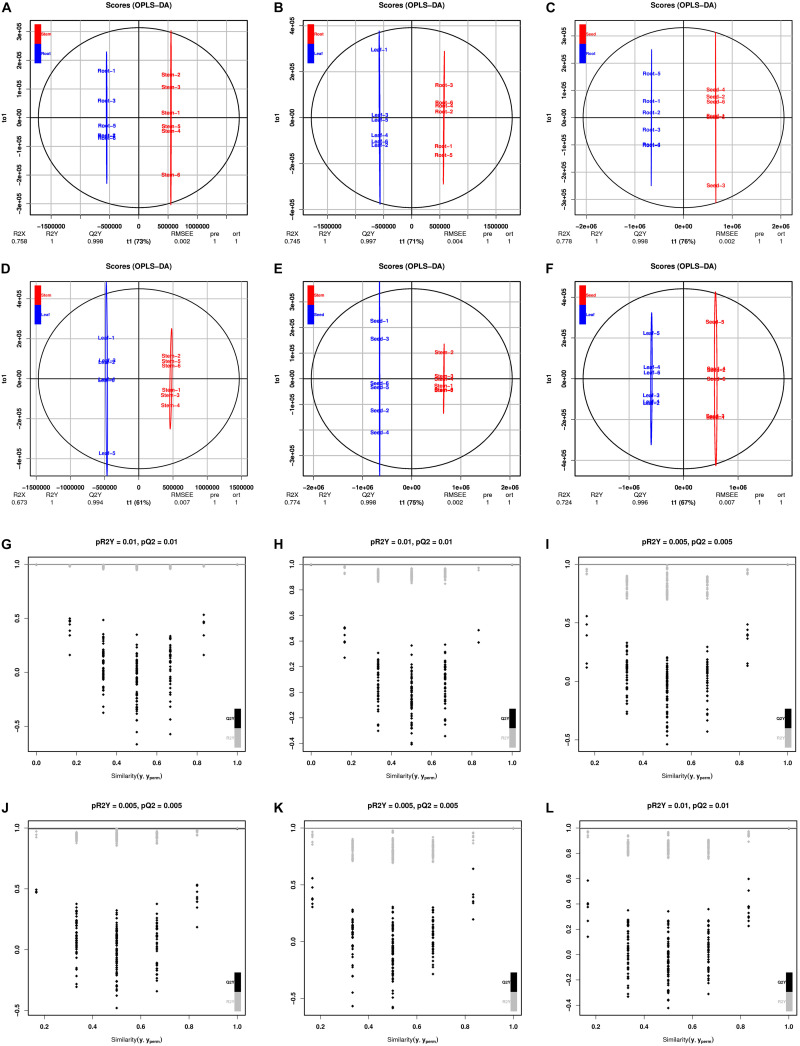
OPLS-DA scores and permutation test. Scores of the OPLS-DA model in **(A)** root vs. stem, **(B)** root vs. leaf, **(C)** root vs. seed, **(D)** stem vs. leaf, **(E)** stem vs. seed, and **(F)** leaf vs. seed. OPLS-DA cross-validation in **(G)** root vs. stem, **(H)** root vs. leaf, **(I)** root vs. seed, **(J)** stem vs. leaf, **(K)** stem vs. seed, and **(L)** leaf vs. seed. R^2^Y and Q^2^ represent the interpretation rate of the model to the Y matrix and the prediction ability of the model, respectively. Q^2^ > 0.9 indicates an excellent predictive model. Permutation test produces a distribution of R^2^’ and Q^2^’ values. A reliable model should yield significantly larger R^2^ and Q^2^ values than R^2^’ and Q^2^’ values generated from random models using the same dataset. Gray and black points represent R^2^’ and Q^2^’ values, respectively.

The PCA revealed that the metabolites of these organ samples were well separated along PC1, thus organ-specific metabolism of *H. glomeratus* was clear (Figure 4). Compared with PCA, OPLS-DA can maximize the distinction between treatments and is more advantageous in identifying differential metabolites due to incorporating an orthogonal signal correction filter into a PLS model. Here, the R^2^ and Q^2^ values all exceeded 0.99 for root vs. stem, root vs. leaf, root vs. seed, stem vs. leaf, stem vs. seed, and leaf vs. seed (Figure 5). Furthermore, the OPLS-DA model was verified by permutation test using 200 alignment experiments. The R^2^’ and Q^2^’ values after replacement were less than the corresponding R^2^ and Q^2^ values of the original model (Figure 5). These results verify that the model could be used to screen differential metabolites with VIP ≥ 1 and *t*-test *P* < 0.05 between two organs.

**FIGURE 6:**
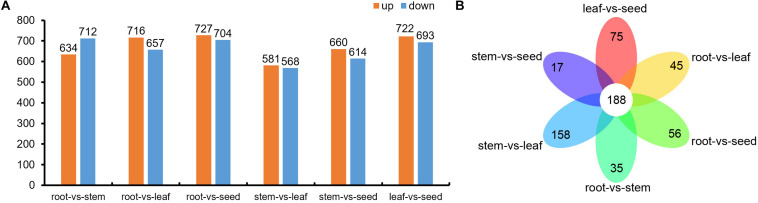
**(A)** Differential metabolites in root vs. stem, root vs. leaf, root vs. seed, stem vs. leaf, stem vs. seed and leaf vs. seed comparisons and **(B)** Venn diagram of the differential metabolites in the root vs. stem, root vs. leaf, root vs. seed, stem vs. leaf, stem vs. seed, and leaf vs. seed comparisons.

Compared to root, a total of 1,346 (634 up-regulated; 712 down-regulated), 1,373 (716 up-regulated; 657 down-regulated) and 1,431 (727 up-regulated; 704 down-regulated) differential metabolites were identified in stem, leaf, and seed, respectively. Analogously, compared to stem, there were 1,149 (581 up-regulated; 568 down-regulated) and 1,274 (660 up-regulated; 614 down-regulated) differential metabolites in leaf and seed, respectively. Furthermore, there were 1,415 differential metabolites in the seed, when compared with leaf, with 722 up-regulated and 693 down-regulated (Figure 6 and Supplementary Table 3). Venn diagram analysis showed 188 common and 35, 56, 45, 158, 17, and 75 specific differential metabolites between root vs. stem, root vs. leaf, root vs. seed, stem vs. leaf, stem vs. seed, and leaf vs. seed, respectively (Figure 6).

### Analysis of Differential Metabolites Among Organs

**FIGURE 7:**
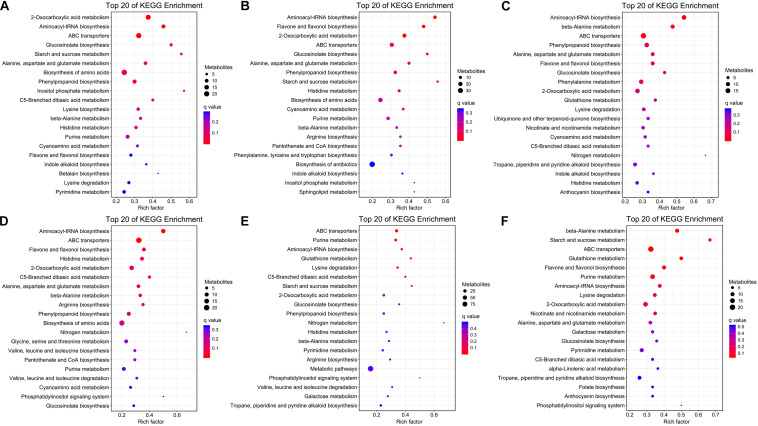
KEGG pathway enrichment analysis of differential metabolites in root vs. stem **(A)**, root vs. leaf **(B)**, root vs. seed **(C)**, stem vs. leaf **(D)**, stem vs. seed **(E)**, and leaf vs. seed **(F)** comparisons.

The KEGG pathway enrichment analysis was conducted to reveal the most important pathways associated with the different organs of *H. glomeratus* at *P* < 0.05 (Figure 7). Compared to root, differential metabolites in stem, leaf, and seed were mainly involved in 2-oxocarboxylic acid metabolism, aminoacyl-tRNA biosynthesis, ATP-binding cassette (ABC) transporters, glucosinolate biosynthesis, alanine, aspartate and glutamate metabolism, phenylpropanoid biosynthesis, and beta-alanine metabolism. Compared to stem, differential metabolites in leaf and seed were mainly involved in aminoacyl-tRNA biosynthesis, ABC transporters, 2-oxocarboxylic acid metabolism, and C5-branched dibasic acid metabolism. Compared to leaf, differential metabolites in seed were mainly involved in aminoacyl-tRNA biosynthesis, ABC transporters and 2-oxocarboxylic acid metabolism (Figure 7 and Supplementary Table 4). Based on the properties of *H. glomeratus*, differential metabolites among organs mainly correlated to the pharmacological effects and response to abiotic stress were further analyzed.

## Discussion

Halophytes can grow on marginal land, withstand high soil salinity and saline water irrigation and are being extensively utilized to identify the genes of salt tolerance, for phytoremediation of soil contaminants and in retrieval of value-added products (Nikalje et al., 2018). There is high interest in the potential applications of halophytes in secondary metabolite production, medicine, food, nutraceuticals, and bioactive compounds. Although *H. glomeratus* has been traditionally used since ancient times as a source of medicinal products and its seeds are rich in unsaturated fatty acids, little is known regarding its nutraceutical or biochemical uses of this species. Metabolomics provides a more comprehensive view of the hundreds of bioactive compounds in plants and for evaluation of their potential value (Banu et al., 2018). In our study, metabolic profiling was carried out in *H. glomeratus* root, stem, leaf, and seed samples using an untargeted GC/MS-based metabolomics approach. To the best of our knowledge, this is the first metabolomics-based report to overview secondary metabolite profiles in *H. glomeratus*. These data represent a potentially valuable data source for the development and utilization of this species.

### Metabolites With Pharmacological Effects Detected in Plants

**FIGURE 8:**
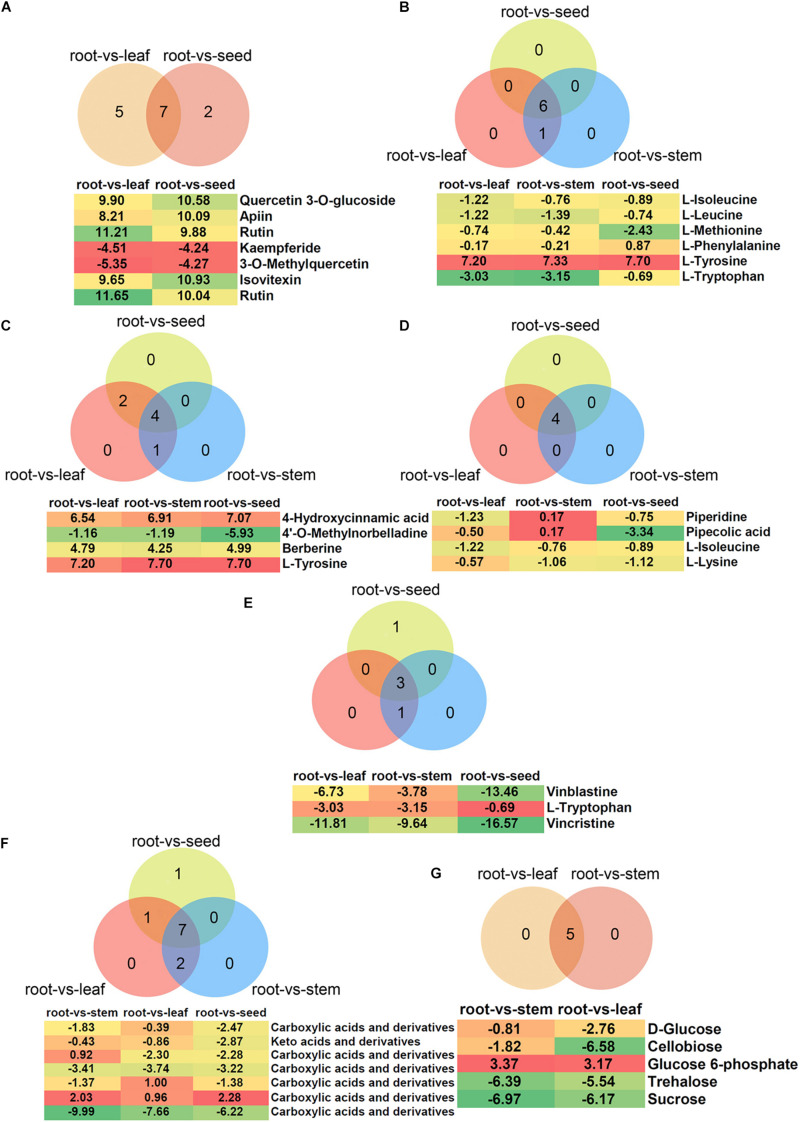
Differential metabolites belonging to **(A)** polyphenols, **(B)** glucosinolates, **(C)** isoquinoline alkaloids, **(D)** piperidine and pyridine alkaloids, **(E)** indole alkaloids, **(F)** amino acids, and **(G)** carbohydrates. Venn diagram shows the overlapping of differential metabolites in different comparisons. The intensity of colors increases with the increasing log_2_ fold-change expression value of root vs. stem, root vs. leaf, root vs. seed, stem vs. leaf, stem vs. seed, and leaf vs. seed comparisons. Values in boxes represent log_2_ fold-change expression value.

In the present study, a total of 35, 25, and 22 metabolites involved in flavonoid, flavone and flavonol and isoflavonoid biosyntheses were detected, respectively (Supplementary Table 3). Of these, the flavone and flavonol biosynthesis pathway was significantly enriched in root vs. leaf (12 metabolites), root vs. seed (9 metabolites), stem vs. leaf (9 metabolites), and leaf vs. seed (10 metabolites), respectively (Supplementary Table 3). Venn diagram analysis showed that seven metabolites overlapped between root vs. leaf and root vs. seed (Figure 8A). The content of quercetin 3-*O*-glucoside, apiin, rutin and isovitexin increased in leaf and seed, while kaempferide and 3-*O*-methylquercetin decreased. Myricetin, laricitrin, kaempferol-3-*O*-rutinoside, kaempferol, and myricetin specifically increased in leaf, while vitexin 2-*O*-beta-D-glucoside specifically increased in seed. These substances have specific functions in prevention of human diseases. For example, quercetin-3-*O*-glucoside is a potential antitumor agent against cancer that acts by inhibiting cell migration induced by tumor-deteriorated growth factors (Lee et al., 2015, 2016). Rutin has demonstrated a number of excellent beneficial health properties, such as prevention and treatment of diseases of the central nervous system, endocrine system, cardiovascular system, cardiovascular system, respiratory system, and excretory system as well as analgesic and antiarthritic activities (Ganeshpurkar and Saluja, 2017). Isovitexin was found to be effective against various cancers through inducing apoptosis and autophagy in liver cancer cells (Ganesan and Xu, 2017).

Glucosinolates and their products play important roles in reducing the risk of carcinogenesis and heart disease (Traka and Mithen, 2009). The KEGG enrichment analyses showed that 14 metabolites were enriched in glucosinolate biosynthesis. Furthermore, the glucosinolate biosynthesis pathway showed significant enrichment in root vs. leaf (seven metabolites), root vs. stem (seven), root vs. seed (six), and stem vs. seed (five) (Supplementary Table 4). Of these differential metabolites, six overlapped among root vs. leaf, root vs. stem and root vs. seed, which were all down-regulated compared to root except for L-tyrosine (Figure 8B).

More than 12,000 alkaloids have been identified in plants and include pyrrolidine, isoquinoline, pyridine, quinoline, indole, and quinazoline (Maione et al., 2016; Rehman and Khan, 2017). Here, a total of 38, 35, and 11 metabolites were identified as involved in isoquinoline alkaloid biosynthesis; tropane, piperidine, and pyridine alkaloid biosynthesis; and indole alkaloid biosynthesis, respectively. However, these metabolic pathways were not significant (*P* > 0.05) enriched in the different comparisons. For isoquinoline alkaloid biosynthesis, five, seven and six metabolites showed significant differences in the root vs. stem, root vs. leaf and root vs. seed comparisons, respectively (Supplementary Table 4). Venn diagram analysis showed that four metabolites overlapped among root vs. stem, root vs. leaf and root vs. seed: 4-hydroxycinnamic acid, 4’-*O*-methylnorbelladine, berberine and L-tyrosine (Figure 8C). The 4’-*O*-methylnorbelladine is regarded as the common biosynthetic precursor for alkaloids in the Amaryllidaceae (Saliba et al., 2015). Berberine plays an important role in traditional Chinese medicine to control blood glucose in type 2 diabetes and treat dysentery and infectious diarrhea (Lau et al., 2001; Yin et al., 2008). Similarly, four metabolites (piperidine, pipecolic acid, L-isoleucine, and lysine) belong to the piperidine and pyridine alkaloid biosynthesis pathway, and overlapped among root vs. stem, root vs. leaf and root vs. seed (Figure 8D). Pipecolic acid is a major metabolic intermediate of L-lysine in the brain, can inhibit food intake and induce sleeping-like behavior in neonatal chicks (Takagi et al., 2001). Furthermore, three metabolites related to the indole alkaloid biosynthesis pathway overlapped among root vs. stem, root vs. leaf and root vs. seed, and were all down-regulated in leaf, stem and seed compared with root (Figure 8E). Vinblastine and vincristine are widely used as potent anticancer drugs and perturb microtubule dynamics by directly binding to tubulin subunits to suppress the self-assembly process of dynamic instability (Castle et al., 2017).

### Metabolites With Response to Abiotic Stress in Plants

Adjusting metabolic status is one important strategy to survive under salt stress. Previous studies indicated that abundance of amino acids and carbohydrates in halophytes may be associated with stress tolerance, especially the accumulation of specific amino acids (Kumari and Parida, 2018; Ravi et al., 2020). Metabolite KEGG pathway enrichment analyses showed that pathways of amino acid metabolism were significantly enriched (Supplementary Table 3). Of these, we found that the arginine and proline, phenylalanine and alanine, aspartate and glutamate metabolism pathways were enriched, which involve free amino acid biosyntheses. Accumulation of free amino acids, such as proline, asparagine and glutamine, has been reported in many studies of plants exposed to salt stress, and these amino acids may contribute to osmotic adjustment (Martino et al., 2003; Keutgen and Pawelzik, 2008; Hildebrandt, 2018). Moreover, the alanine, aspartate and glutamate metabolism pathway were significantly enriched in stem, leaf and seed compared to root; metabolites of carboxylic acids and derivatives and keto acids and derivatives were down-regulated (Figure 8F and Supplementary Table 3). Of course, its rich amino acid composition increases the potential of *H. glomeratus* for supplying pharmaceutical products.

In general, salt stress induces a large increase in carbohydrate pools to deal with osmotic adjustment (Tiago et al., 2016). In this study, many metabolites belonging to carbohydrate metabolism were identified, with the starch and sucrose metabolism pathway enriched in leaf and stem compared to root (Supplementary Table 3). However, D-glucose, cellobiose, trehalose and sucrose were increased in root but not glucose 6-phosphate (Figure 8G). The accumulation of sucrose in xerohalophyte *Atriplex halimus* leaves under high salinity has been attributed to photoprotection of photosystem II (Bendaly et al., 2016). Exogenous application of salicylic acid strongly stimulated the biosynthesis of glucose, mannose, fructose, and cellobiose to alleviate damage due to drought (Zhou et al., 2017). Our data indicate that carbohydrate metabolism may have crucial functions in response to salt and drought stress in *H. glomeratus*.

Lipids are the major component of the cell plasma membrane, and alterations in membrane lipids have crucial roles in response to salinity stress (Hou et al., 2016; Guo et al., 2019). Metabolites involved in lipid metabolism were enriched in KEGG pathway analyses of this species, including in arachidonic acid metabolism (37 metabolites), linoleic acid metabolism (21), biosynthesis of unsaturated fatty acids (20), and glycerophospholipid metabolism (37). However, significant differential expression of lipid metabolites in root, stem, leaf and seed was not detected in our metabolism analysis. This result not only is consistent with *H. glomeratus* being rich in unsaturated fatty acids, especially linoleic acid (Li et al., 2019), but also indicates that lipids acting as very important biomolecules are widely distributed in all tissues of *H. glomeratus* in response to salt stress.

In plants, ABC transporters play an important role in coping with salt stress processes because they can act as exporters as well as importers for transporting a myriad of substrates across biological membranes (Hwang et al., 2016). Our results showed metabolites related to ABC transporters were significantly enriched in root vs. stem (20 metabolites), root vs. leaf (19), root vs. seed (19), stem vs. leaf (12), and stem vs. seed (12) in *H. glomeratus* (Supplementary Table 1). These different metabolites may enhance the ability of *H. glomeratus* to avoid toxins by transporting toxic compounds under salinity and drought stress.

## Conclusion

**FIGURE 9:**
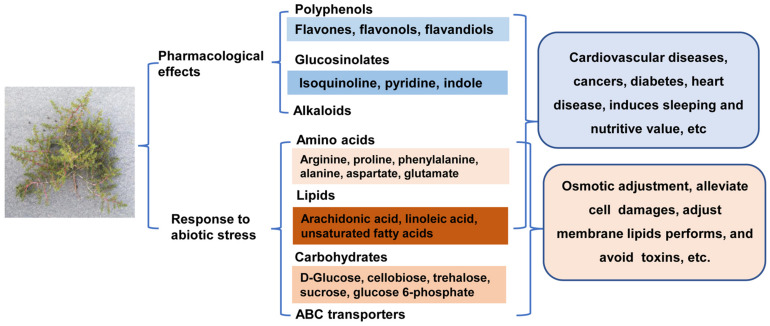
Overview of the certain important metabolites involved in pharmacological effects and response to abiotic stress in *H. glomeratus*.

We investigated the variability of the metabolites of root, stem, leaf, and seed in halophyte *H. glomeratus* using an untargeted metabolomics approach. The results showed that differential metabolites in stem, leaf, and seed were mainly involved in 2-oxocarboxylic acid metabolism, aminoacyl-tRNA biosynthesis, ABC transporters, glucosinolate biosynthesis, phenylpropanoid biosynthesis, beta-alanine metabolism and alanine, aspartate and glutamate metabolism compared to root. Notably, important metabolites with pharmacological effects and that respond to abiotic stress were successfully identified in *H. glomeratus* (Figure 9). However, it should be emphasized that the molecular features of 10,393 metabolites were still unknown. These results provide reference for evaluating the medical utilization value and understanding the salt-tolerance mechanism of this species. Future studies should confirm the results obtained in this work and compare different classes of metabolites presented in the same tissue by analyzing *H. glomeratus* plants collected from different regions of China. Then, a number of candidate metabolites with pharmacological effects will be extracted and characterized through a series of pharmacological experiments. We hope that our findings will open a new field to accelerate the future development of *H. glomeratus*.

## Data Availability Statement

The original contributions presented in the study are included in the article/Supplementary Material, further inquiries can be directed to the corresponding author/s.

## Author Contributions

JW and KY carried out the metabolomic analysis and drafted the manuscript. LY, ZM, ES, BL, and XM participated in material culture and performed the statistical analysis. XS and HW conceived of the study and participated in its design. HW, CL, and YM helped to draft the manuscript. All authors read and approved the final manuscript.

## Conflict of Interest

The authors declare that the research was conducted in the absence of any commercial or financial relationships that could be construed as a potential conflict of interest.

## Footnotes

1 http://www.hmdb.ca

2 http://metlin.scripps.edu

3 http://www.massbank.jp/

4 http://www.lipidmaps.org

5 https://www.mzcloud.org

6 http://www.r-project.org/

## Supplementary Material

Supplementary Table 1 Summary of all metabolites detected in *H. glomeratus*.

Supplementary Table 2 Category summary of all identified metabolites in *H. glomeratus*.

Supplementary Table 3 Summary of differential metabolites in root vs. stem, root vs. leaf, root vs. seed, stem vs. leaf, stem vs. seed and leaf vs. seed comparisons.

Supplementary Table 4 Detailed information of KEGG pathway enrichment analysis of differential metabolites in root vs. stem, root vs. leaf, root vs. seed, stem vs. leaf, stem vs. seed and leaf vs. seed comparisons.
